# Editorial: Rising stars in biofilms 2022

**DOI:** 10.3389/fcimb.2023.1169998

**Published:** 2023-03-03

**Authors:** Chelsie E. Armbruster, Ângela França, Almagul Kushugulova, Renan M. Mauch, Alberto J. Martín-Rodríguez

**Affiliations:** ^1^ Department of Microbiology and Immunology, Jacobs School of Medicine and Biomedical Sciences, University at Buffalo, Buffalo, NY, United States; ^2^ Biofilm Research Laboratory Rosario de Oliveira, Center of Biological Engineering, University of Minho, Braga, Portugal; ^3^ Microbiome Laboratory, Center for Life Sciences. National Laboratory Astana, Nazarbayev University, Astana, Kazakhstan; ^4^ Unit of Airway Inflammation, Biomedical Centre, Faculty of Medicine, Lund University, Lund, Sweden; ^5^ Department of Microbiology, Tumor and Cell Biology, Karolinska Institute, Solna, Sweden

**Keywords:** biofilms, bacterial infection, mycoses, antibiotic resistance, multidrug-resistant bacteria, *Candida* spp., *Pseudomonas aeruginosa*, dental infection

The emergence of antibiotic-resistant bacteria, including globally-disseminated multidrug-resistant strains, has become one of the most alarming threats for human health. Fundamentally originated by an inadequate use of antibiotics in clinical and non-clinical settings, infections caused by multidrug-resistant bacteria are currently responsible for around two million deaths each year and are estimated to be the cause of death of as many as 10 million people yearly by 2050 (O'Neill, 2016). In parallel, mycoses, which are often concomitant with bacterial infections, have been historically neglected despite being the fifth largest cause of death and imposing a huge burden on healthcare systems worldwide (Rodrigues and Nosanchuk, 2020).

Biofilm formation is a common characteristic of numerous bacterial and fungal species of clinical relevance. In bacteria, biofilm formation involves the production of an extracellular matrix mainly composed of exopolysaccharides, proteins, and nucleic acids, whose architecture and dynamics are impressive in many ways. Biofilms protect pathogens from the action of drugs and the immune system and facilitate their survival in hostile environments such as low oxygen, nutrient deprivation, and the host inflammatory response. Within biofilms, the so-called “persisters”, that is, cellular subpopulations with reduced metabolic activity, contribute to bacterial resilience and infection relapse while remaining largely immune to the action of most antibiotics (Flemming et al., 2016).

Much progress has been achieved in bacterial biofilms research over the last years, using a plethora of models mimicking infection such as cystic fibrosis, wound, dental, osteomyelitis, endocarditis and medical-device related infections (Banerjee et al., 2020). However, substantial efforts are still needed to develop therapeutic strategies to tackle the challenges posed by biofilms, including an improved understanding of their complex physiology and dynamics. The new generation of scientists has a lot to contribute in this regard. In the present Research Topic, we gather four studies led by early-career scientists that bring new insights and advances to biofilm research.

In the bacteriology field, LuTheryn et al. developed a sophisticated *in vitro* model of wound infection to demonstrate the ability of ultrasound-responsive gas microbubbles to disrupt *Pseudomonas aeruginosa* biofilm, reducing the number of culturable bacteria by 26.9-99.8%. In the mycology field, two studies complement each other by making an in-depth analysis of the poorly understood biofilm development in *Candida* species. Atiencia-Carrera et al. used a variety of assays to demonstrate an increased biofilm build-up in *Candida tropicalis* compared to *Candida albicans*. Kärkkäinen et al. used fluorescent probes to visualize the yeast and hyphal forms of *C. albicans* and showed intracellular amyloid aggregates within a subpopulation of cells in the biofilm, as well as polymorphic forms of these aggregates between yeast and hyphal cells. These two studies provide reliable methods to understand the physiology of *Candida* spp. biofilms and lay the ground for the development of diagnostic methods and drug strategies targeting them. Finally, Negrini et al. scrutinize both bacterial and fungal kingdoms and, using a model mimicking dental infection, show a broad diversity of bacterial and fungal aggregates interacting with each other and varying in size, structure, pH, and spatial organization, depending on the types of dietary sugar found in saliva samples. Notably, a sucrose and a sucrose-starch combination resulted in biofilms with higher biomass and acidogenicity with a complex network of bacterial and fungal cells, when compared to the ones formed in saliva without sugar or with glucose and fructose only. These data suggest that these compounds have an important role in the development of severe early childhood caries.

Taken together, the contributions of these rising stars to the biofilm field provide not only novel knowledge on bacterial and fungal biofilm development and their interactions, but also potential therapeutic and preventive approaches that can be seized in several disease models and possibly in connection with current technologies, such as artificial intelligence, allowing in-depth studies of the mechanisms of biofilm formation that can give rise to new theories. The topic is definitely of special interest to medical doctors, clinical practitioners and dentists.

## Author contributions

All authors have read, reviewed and approved the final text and contributed equally to this editorial.

## References

[B1] O'Neill J. (2016). Tackling drug-resistant infections globally: Final report and recommendations. Review of Antimicrobial Resistance (AMR). Government of the United Kingdom. Available at: https://amr-review.org/sites/default/files/160518_Final%20paper_with%20cover.pdf (Accessed: February 21, 2023).

[B2] Banerjee D. Shivapriya P. M. Gautam P. K. Misra K. Sahoo A. K. Samanta S. K. (2020). A review on basic biology of bacterial biofilm infections and their treatments by nanotechnology-based approaches. Proc. Natl. Acad. Sci India Sect B Biol. Sci. 90, 243–259. doi: 10.1007/s40011-018-01065-7

[B3] Flemming H.-C. Wingender J. Szewzyk U. Steinberg P. Rice S. A. Kjelleberg S. (2016). Biofilms: An emergent form of bacterial life. Nat. Rev. Microbiol. 14, 563–575. doi: 10.1038/nrmicro.2016.94 27510863

[B4] Rodrigues M. L. Nosanchuk J. D. (2020). Fungal diseases as neglected pathogens: A wake-up call to public health officials. PloS Negl. Trop. Dis. 14, e0007964. doi: 10.1371/journal.pntd.0007964 32078635 PMC7032689

